# A qualitative interview study exploring barriers and facilitators to uptake of measles vaccination among healthcare workers at a London hospital

**DOI:** 10.3389/fpubh.2025.1621699

**Published:** 2025-09-26

**Authors:** Nikki Heinze, Louise E. Smith, Carmel Curtis, Dale Weston, Jasmin Islam, G. James Rubin

**Affiliations:** ^1^Institute of Psychiatry, Psychology and Neuroscience, King’s College London, London, United Kingdom; ^2^NIHR Health Protection Research Unit in Emergency Preparedness and Response at King’s College, London, United Kingdom; ^3^UK Health Security Agency, London, United Kingdom; ^4^King’s College Hospital NHS Foundation Trust, London, United Kingdom

**Keywords:** measles, MMR, healthcare workers, vaccination, vaccine hesitancy

## Abstract

**Introduction:**

Healthcare workers (HCW) are at increased risk of measles due to their occupational exposure. Yet, there is evidence of low vaccination rates, inadequate immunity among this group, and many do not know their vaccination status. The aim of this qualitative study is to explore barriers and facilitators to measles vaccination and reasons why some HCW do not know their vaccination status.

**Methods:**

We conducted 23 online semi-structured interviews with HCW recruited from a teaching hospital in London. HCW were eligible to participate if they had direct patient contact, had not had measles, and were either (a) unsure of their vaccination status, (b) unvaccinated, (c) partially vaccinated, or (d) vaccinated after joining the hospital. We used framework analysis to identify themes and subthemes.

**Results:**

Facilitators to measles vaccination included protection of self and others, being prompted and pragmatic considerations such as being required to be vaccinated for work. Barriers included the accessibility of vaccination, concerns about vaccine safety, and low perceived risk of and from measles. Fractured vaccination records and a lack of perceived importance of measles vaccination may contribute to some HCW not knowing their vaccination status.

**Conclusion:**

Making vaccination accessible, increasing knowledge and awareness of measles and measles vaccination, and prompting those who require vaccination may support vaccination decisions. A central, easy-to-access App or portal which sends reminders for boosters may reduce the number of HCW who are unsure of their vaccination status.

## 1 Introduction

In 2024, England recorded 2,911 laboratory confirmed cases of measles, the highest number since 2012 (1). Recent measles outbreaks, including in the UK, have partly resulted from nosocomial transmission (2–13). This may be facilitated by the presence of a large number of, often immunocompromised, people in a confined space, the high reproduction rate of the measles virus, its ability to remain airborne for protracted periods of time, and delayed diagnosis of measles due to infected individuals attending hospital before the onset of the characteristic rash (13–15). The success of previous measles vaccination campaigns means that many healthcare workers (HCW) have less experience recognizing the symptoms, leading to delayed diagnosis and management of the disease (13).

Measles vaccination has formed part of the UK childhood immunization schedule since 1968 (16). UK children should have received both doses of the measles, mumps and rubella (MMR) vaccine before the age of five (16), but coverage among 5-year-olds has been lower than the 95% target since 2018–2019 (17). The UK Health Security Agency recommends, but does not mandate, measles vaccination for HCW (18). Compared to the general population, HCW may be 2–19 times more likely to contract measles due to their increased risk of contact with cases accessing healthcare (19, 20). In measles outbreaks in Italy and France, HCW accounted for 7–19% of cases (12, 21, 22). The relative success of the measles vaccine in reducing outbreaks may have resulted in complacency, while the publication in 1998 of a now-discredited article linking the MMR vaccine to autism (23, 24) impacted confidence in the vaccine. A survey of 133 HCW in Wales found that 20.5% were not vaccinated (25). Overall, between 3.3% and 26.6% of HCW in the UK may lack immunity to measles either through vaccination or infection, and this is more prevalent among younger HCW (26, 27).

Between 9% and 35% of UK HCW are unsure of their measles vaccination status (25, 26), while evidence from measles outbreaks has highlighted that the vaccination status of HCW is not always recorded (11, 22, 28). A survey of 104 hospital trusts in England found that 85 had immunization policies and 94 offered the MMR vaccine to their staff, but only 48 recorded MMR staff vaccination information on a central database and 16 recorded staff eligibility for the MMR vaccine (29). Poor record keeping is problematic because efficient identification of unvaccinated staff members is needed to reduce transmission during an outbreak.

To design effective interventions to facilitate measles vaccination among UK HCW, a clear understanding of the barriers and facilitators to uptake is required. We report findings from a qualitative study using semi-structured interviews to explore:

1. What are the barriers and facilitators to uptake of measles vaccination among UK HCW?
2. Why do some HCW not know their vaccination status?

## 2 Materials and methods

### 2.1 Participants

HCW were recruited from one hospital in London (the Trust), which recently experienced an outbreak of measles. Participants were recruited using convenience sampling. Recruitment materials were disseminated through the staff intranet, bulletins, and clinical teams. Members of the research team presented at a meeting of the Equality and Diversity Group to raise awareness of and support for this research.

HCW were eligible to take part if they were aged 18 and over, had direct contact with patients, had not had measles in the past and: did not know if they had been vaccinated (unsure) to address research question 2, had not had the vaccine (unvaccinated), had received only 1 dose of the MMR vaccine (1 dose) or had received the MMR vaccine after joining the Trust (2 doses after joining the Trust) to explore research question 3. The questions used to screen participants are available as Supplementary material. Due to the potentially sensitive nature of disclosing vaccine status in a work environment, we decided to allow participants to self-report their vaccination status. However, this means that vaccination status was not objectively verified. Of the 36 HCW who contacted us, 13 (36.1%) did not respond after being sent a participant information sheet or were not eligible to take part. A total of 23 HCW took part in the interviews. One participant subsequently disclosed that they had had measles as a child, but the interview was continued to gain their insights into the health screening process and general attitudes toward vaccination. Research suggests saturation can be reached with 9–17 interviews (30) and we did not discover any new themes in the final interviews indicating that saturation had been reached.

### 2.2 Materials

We used a semi-structured interview schedule to assess, among other things, the reasons for not knowing one’s vaccination status, reasons for not being fully vaccinated, barriers and facilitators to measles vaccination, knowledge and attitudes toward the measles vaccine, and knowledge of measles (Supplementary material). The interview also explored experiences of the occupational health (OH) pre-employment screening process and measles campaigns.

### 2.3 Procedure

All participant liaison, data collection and analysis were conducted by researchers unaffiliated to the Trust. Interested HCW contacted us, received a participant information sheet and had a brief call on Microsoft Teams to answer any questions, check eligibility and arrange an interview on Microsoft Teams. Participants completed separate demographics questionnaires and a consent form before the interview. Interviews were video or audio recorded depending on the participant’s consent. Participants were sent a £40 Amazon voucher as a thank-you for their time. Interviews were conducted between September and December 2024. The interviews lasted between 25 and 54 min (Mean = 40 min).

Ethical approval was obtained from the King’s College London Research Ethics Committee (LRS/DP-23/24-42520) and the Health Research Authority (IRAS ID 344919).

### 2.4 Analysis

Audio files were transcribed by a professional transcription company. The analysis followed the five stages of framework analysis (31). After familiarization with the data (stage 1), a thematic framework was identified informed by the topic areas (barriers/facilitators, knowledge of measles, exposure to measles, etc.) explored in the interview (stage 2). The transcripts were then uploaded into NVIVO 14 and coded or ‘indexed’ using the framework (stage 3). The data were charted into an Excel spreadsheet along with quotes from the transcripts (stage 4). The charted data were mapped and interpreted against the research questions (stage 5). The five stages of framework analysis reflect the six phases outlined by Braun and Clarke for thematic analysis (32, 33): familiarizing yourself with your data (phase 1), generating initial codes (phase 2), searching for themes (phase 3), reviewing themes (phase 4), defining and naming themes (phase 5), and producing a report (phase 6).

## 3 Results

Table 1 provides an overview of the characteristics of the 23 participants, who were all female.

**Table 1:** Participant characteristics (*N* = 23).

Variable	Category	*n*	%
Age	25–34	7	30.4
	35–44	11	47.8
	45–54	3	13.0
	55–64	2	8.7
Ethnicity	Asian/Asian British	8	34.8
	Black/Black British/Caribbean or African	5	21.7
	White/White British	10	43.5
Residence until the age of 5	United Kingdom	11	47.8
	Africa	3	13.0
	Asia	6	26.1
	Europe	3	13.0
Time spent working at the Trust	1–2 years	2	8.7
	2–5 years	5	21.7
	More than 5 years	16	69.6
Vaccination status	Not sure	10	43.5
	Unvaccinated	2	8.7
	1 dose	7	30.4
	2 doses^1^	4	17.4

1 All four received at least one dose after joining the Trust.

Percentages for each category may not total 100% due to rounding. Due to low frequencies for some categories, higher level categories are shown for ‘Ethnicity’ and ‘Residence until the age of 5’ variables.

### 3.1 Barriers and facilitators to uptake of measles vaccination among HCW

**Figure 1:**
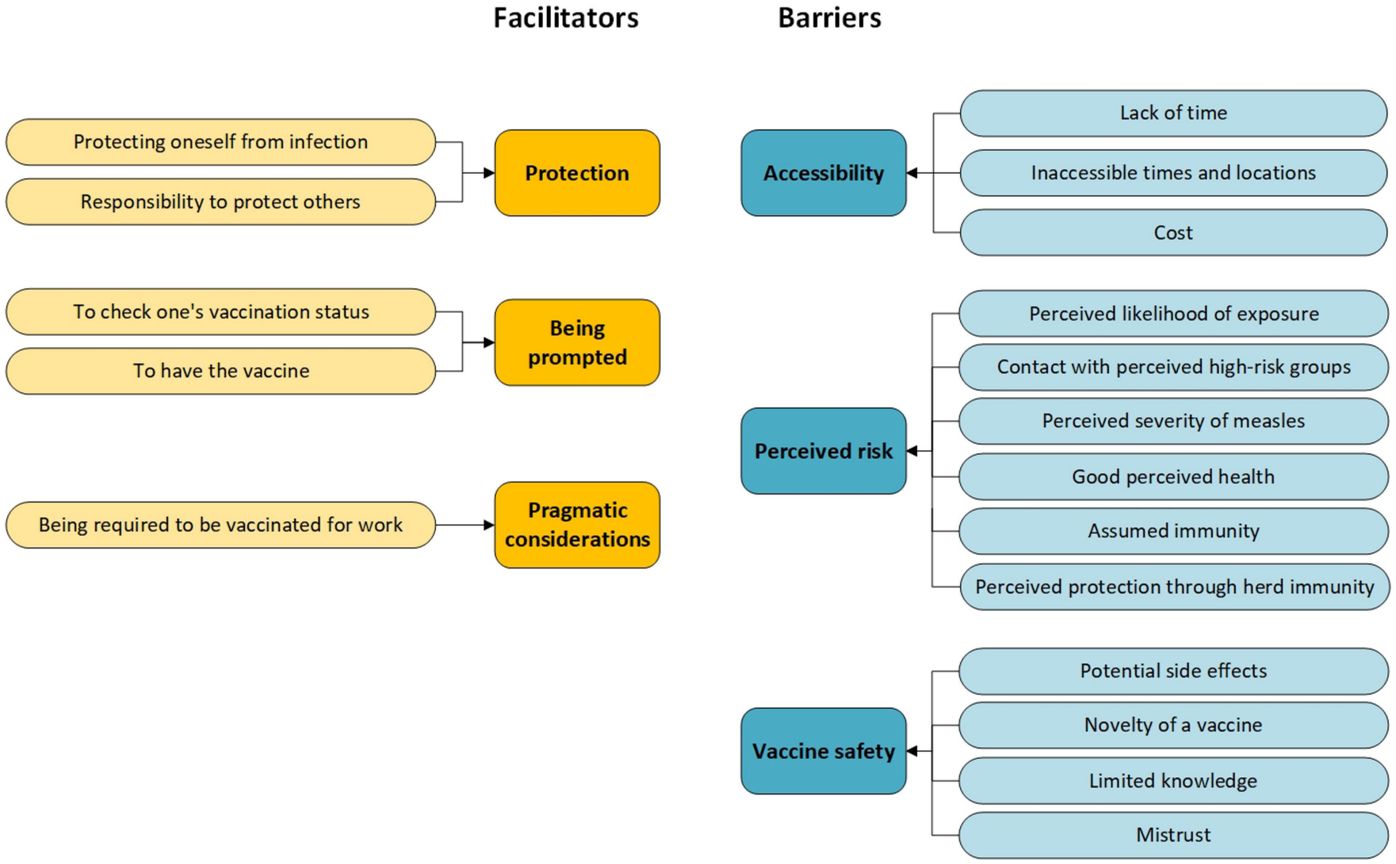
Overview of facilitators and barriers to measles vaccination among HCW.

Participants were generally in favor of vaccination. Most would have the measles vaccine if they were asked to do so, but not always without reservations. Figure 1 provides an overview of the six key themes that we identified in our interviews, divided into the motivators and barriers for measles vaccination. In this results section, the names of subthemes are indicated in the text in italics.

#### 3.1.1 Protection

*Protecting oneself from infection* and illness was a common motivation for having the vaccine.

*Mainly to protect myself, if there was an outbreak and it was going to prevent me from getting it, I think I’d be very inclined to having it done.* HCW08, Unsure.

However, many also described a sense of *responsibility to protect others* around them from infection, including family and patients, particularly if immuno-compromised, and the wider community.

*Just to protect myself and my [child] really and also the patients that I would be seeing […] I’d feel really guilty if I was to pass it on to somebody.* HCW02, 1 dose.

*Vaccination is not just about protecting yourself; it’s about protecting society.* HCW19, Unsure.

#### 3.1.2 Being prompted

Several participants suggested that being prompted *to check one’s vaccination status* or *to have the vaccine* would motivate them to do so.

*I think we all need a prompt when you are working in healthcare, to do something for yourself. If it’s for your patient, you are very proactive, you’ll go above and beyond to do it. But when it comes to protecting yourself, we all need a nudge for us to really go ahead and do it.* HCW17, Unsure.

*I’ve got to say from the emails and us doing this today, I will go away and find out, because you know when you just think I should know, I definitely should know. Now, if we do this again in two years I might have forgotten again because I do not know what else I’ve had been vaccinated against.* HCW27, Unsure.

The use of prompts to inform people of the need for a second dose of the vaccine and to make HCW feel valued was also raised.

*We get sent a reminder when your mandatory training is expiring, I’m sure they can create something like that. […] And it will make you feel a little bit more valued as well and the Trust is thinking about the health and wellbeing of their members, their employees.* HCW16, Unsure.

#### 3.1.3 Pragmatic considerations

Several participants described receiving measles vaccines because they felt they were *required to be vaccinated for work*. Some would not have had the vaccine if they felt that they had a choice.

*Well, it was literally like I needed to take the job obviously and I could not work without having the vaccination. So, it was literally like you have to have the vaccination if you want to work in this line of job. […]. [Could I ask, if you felt that you had had more of a choice, what would you have decided to do?] I’d have probably left it at that point*. HCW04, 2 doses after joining the Trust.

While some participants acknowledged that a measles vaccine mandate would encourage them to be vaccinated, others suggested that HCW should be involved in vaccination decisions, by providing sufficient information and space to discuss concerns beforehand, while taking into consideration the time constraints of HCW.

*So, if they want to improve [the process at the Trust], they should try and create better awareness, engage the staff properly, let them understand, and let them be part of the process.* HCW30, 2 doses after joining the Trust.

#### 3.1.4 Accessibility

A recurring theme across the interviews was the *lack of time* HCW had at work, with many prioritizing their patients over themselves, which created a barrier to finding out their vaccination status and having vaccines.

*In my role, because I’m clinical, quite a lot is usually patient-facing, so you have to actively walk away from the thing that’s important to that person right then to do something preventative for yourself.* HCW27, Unsure.

*When the measles came up, I do not know if I’m vaccinated, I do not know if I can get an appointment in occupational health easily to see if I am vaccinated or if I’m immune against it. So, we kind of, like, “Okay,” we brush it past because, again, when we are at work, we are super busy, we do not have the time to sit and think as well.* HCW17, Unsure.

Perhaps unsurprisingly, *inaccessible times and locations* were the most cited ways to make it easier for HCW to have the vaccine.

*Obviously, if it was available on site at work, that would be great, and then I guess flexibility in terms of timing when we could go and get it because working in healthcare your time is never your own so finding a gap to actually go and get a vaccine can sometimes be tricky.* HCW36, Unsure.

Accessibility may also extend to *cost*. Although unvaccinated HCW have access to free MMR vaccination through their hospital’s OH team, some were unaware of this and listed cost as a potential barrier.

*I think cost wise would affect me. If it was really expensive, I may not prioritise it as I should, or it might take longer for me to get it.* HCW23, 1 dose/Unsure.

#### 3.1.5 Perceived risk

The *perceived likelihood of exposure* to measles was a consideration for participants and varied from person to person.

*Considering that I’ve been doing this job for [more than ten] years and not come across it, I do not think I will be exposed. I do not know.* *Who knows*? HCW35, Unvaccinated.

*I would say quite likely because our patients, the treatments that they go through and because [of their condition], almost 90% of our patients are immunocompromised, so they are very prone to catching whatever infections are out there.* HCW17, Unsure.

For instance, an outbreak may act as a motivator to be vaccinated.

*If MMR was on the rise in the local community, then yes, I probably would, just being in the medical profession.* HCW28, 1 dose.

For some, risk was determined by their *contact with perceived high-risk groups*. For instance, some participants associated measles with children, lowering their perceived risk if they had little contact with children. But there was disagreement if the risk of catching measles was higher in outpatient or inpatient settings.

*Now that I work in outpatients it’s probably higher, because there’s kids and stuff. But before, on the wards, you are protected, you are quite isolated, but there’s still different sorts of infections.* HCW06, 2 doses after joining the Trust.

*… a lot of my interaction with patients is in an outpatient setting. Very rarely, very, very rarely am I seeing a patient that’s been hospitalised. […] I wonder if I do contract measles, it will be from an inpatient.* HCW03, Unsure.

Concern about the *perceived severity of measles* varied. Higher concern often related to spreading the infection to family and patients.

*If I did have it, I would probably be… well, very worried about it because I would not want [my child] to have it or would not want my husband or I guess any other family member to get it either, to be fair. And then, because I also work with patients who are medically compromised, yeah, I would not want to spread it to other people.* HCW13, Unsure.

*Good perceived health* and confidence in their own immune system reduced this concern.

*But in my view, right now, if I’m honest with you, it’s not something that I will go out of my way to go and get vaccinated for, if that makes sense, because I feel like I have my health at the moment. I feel like I’m quite healthy, if I’m quite honest.* HCW21, Unsure.

Several participants gave *assumed immunity* from exposure or prior vaccination as reasons for not having been vaccinated.

*I sort of felt that maybe I would have some sort of immunity, like an acquired immunity from being exposed to people in the community […].* HCW04, 2 doses after joining the Trust.

Finally, perceived risk may also be impacted by the perception that everyone else is vaccinated, resulting in *perceived protection through herd immunity*.

*I think, not necessarily for me, but I do know some people who would consider, if everyone else around me is vaccinated, do I really need to have the vaccine?* HCW28, 1 dose.

#### 3.1.6 Vaccine safety

Along with perceived risk, vaccine safety, specifically *potential side effects*, both short- and long-term, were a main concern for participants.

*So, things like how it’s going to affect me, is it going to make me unwell maybe that weekend, or is it going to make me unwell long-term, that’s the main thing. Am I going to now get measles. Yes, I think just the getting unwell thing, that was bugging me.* HCW06, 2 doses after joining the Trust.

*The long-term side effects of the vaccine itself. But I think I would probably be more swayed to have it, even with knowing the side effects. Because also I’ve not really had any adverse reactions to any vaccine that I’ve had, so that’s probably why I am still brave to take any vaccine.* HCW35, Unvaccinated.

Almost half (*n* = 10) of the participants spontaneously referred to the link between MMR and autism, although several referred to the link as a ‘*myth’* or ‘*old wives’ tale’*.

*There’s a lot of myths out there, and I have not really taken the time to look is it true or not, but I’ve heard things about autism being linked to the vaccine, and other conditions as well, but I do not think it is really.* HCW06, 2 doses after joining the Trust.

*I know back in the day, there was a lot of talk about the MMR vaccine, autism, and the link between those. I would not necessarily say that that would put me off. I think being in this role, I was very pro vaccination, especially the flu vaccines, Covid vaccines, things like that. But I did not have a negative view towards it, despite the literature. But it also sounded a bit like an old wives’ tale.* HCW28, 1 dose.

However, two participants described how the risk of autism informed their vaccination decisions for their children.

*So, it is mainly the link to autism which I know sparked the initial concerns. For my second [child], we ended up doing singular vaccines. My first [child] wasn’t immunised for measles at all.* HCW12, Unsure.

The *novelty of a vaccine* may impact perceptions of its safety. Concerns about the speed with which the COVID-19 vaccine was developed, and the lack of data on long-term consequences contrasted with the perception that measles vaccines had been around long enough to yield sufficient evidence of safety.

*And again, it’s hypothetical, but you are still seeing, with newer vaccines, also with the MMR, if it were to be compulsory, you do not necessarily know which brand you are getting, how long it’s been in the market for, and if it were a relatively new vaccine, or a new formulation of the vaccine, we do not necessarily know the impact it has in years to come.* HCW28, 1 dose.

Most participants reported having *limited knowledge* of measles and the measles vaccine, possibly prompted by the lack of exposure to measles in their personal and professional lives, which could impact vaccination decisions.

*I will put my hands up, I’m just completely ignorant when it comes to measles or MMR. But I think that’s just based on lack of exposure to it.* HCW03, Unsure.

*[A private healthcare company] asked me to have the second MMR vaccine, and I did not even know it was a thing until then.* HCW06, 2 doses after joining the Trust.

Only six of the 23 HCW in this study were aware of any local Trust communications about measles. Some participants described learning more about measles through their children.

*I think that my memory has been refreshed from when my [child] had it, they tended to tell me to look out for a rash and stuff like that…* HCW04, 2 doses after joining the Trust.

*[And how much do you feel you know about the measles vaccine?] I know a little bit about it because, as I say, from my children and things like that.* HCW16, Unsure.

Several participants wanted more information about the vaccine and measles to enable them to make an informed decision.

*To be honest, when we are at work, the clinical side of things, the only people that really manage to catch the emails [from the Trust] are staff that have access to a computer. Most of the time the computer on clinic is completely in use with clinical activity […] You tend to hear from peers or word of mouth. With a poster you do not actually have to log on or make sure that a computer is even free for you to be able to see it. Also, we have lots of age differences here which for old people technology is not always the way forward and they could be a group that are at risk.* HCW04, 2 doses after joining the Trust.

A small number of participants raised the issue of a general *mistrust* in healthcare and ‘big pharma’.

*There’s a lot of mistrust against healthcare people unfortunately. Even within healthcare workers. I know myself, when I was off from [unit], and it was actually time to take the vaccine, and everybody in my family was like ‘you do not want to take it, you do not know what it is’,* HCW06, 2 doses after joining the Trust.

### 3.2 Why do some HCW not know their vaccination status?

Ten of the 23 HCW did not know their vaccination status. Thematic analysis resulted in two key areas to explain this.

#### 3.2.1 Fractured vaccination records

Knowing one’s vaccination status relies on access to up-to-date records but accessing records can be challenging:

*I’m not sure whether they gave me the measles vaccine. […] I do not even know where to check. Maybe my GP would have it. […] The hepatitis one, I think it was through the Trust. They wanted us to have it before we started, or before we started seeing patients. I think so. I do not know. I mean, we are talking [more than ten] years ago now. I have a hard time remembering what I did last week.* HCW35, Unvaccinated.

Fragmentation in vaccination recording and a reliance on parental recall can make it challenging to get an accurate vaccine history.

*The only person that I can think of is my mum if I was to ask somebody, my dad would not have a clue. But I do not know whether there are any records […]. I do not know whether [existing online records] hold that information back that far or whether my GP would know.* HCW11, Unsure.

In addition to routine immunizations administered by GPs, at school and recorded in a paper booklet during childhood, HCW described receiving vaccines for travel or work, privately, through their GP, work, or pharmacists. Participants who had immigrated to the UK or had lived abroad described particular difficulties in collating records.

*I think it would have been difficult because I’m born in [country], trained in another part of [same country], and then moved to [other country], moved here, I think it would be very sketchy to put all the information together. But in view of that, I’ve always been careful with my children to make sure that their vaccination history is very, very… in one place. But I think, personally, I do not know if I would be able to collect all the information and how to go about it.* HCW16, Unsure.

Where participants had obtained their vaccination records, these were not always complete, and paper or electronic copies were easily lost or misplaced.

*I did have a copy from my original Trust. I think I did, but then I do not know. I’ve moved so much, so I do not really know where they are now. But then I might be lucky, I might find it at some point when I’m looking for something else.* HCW35, Unvaccinated.

Moreover, staff vaccination records may be outdated and/or incomplete if existing records are not regularly reviewed and updated, incomplete or missing information is actively sought and those transferring from non-patient-facing roles into patient-facing roles are not invited to undergo the OH screening.

*[Did you update your vaccination status after you had the second dose through a private company?] Not with [the Trust], no. [So, they do not know] No, [the Trust] does not know. Maybe it’s because nobody asked me.* HCW06, 2 doses after joining the Trust.

Many welcomed the idea of an app where vaccination records could be stored for quick access, particularly if reminders for boosters were built into the app.

*That would be nice actually, because it’s more accessible, it’s more updated, so we can keep a track on ourselves. And then if you get the notification ‘you are due for this vaccination’, let us go and take it then. It would be very nice actually.* HCW07, 1 dose.

However, concerns about the security of their data, accessibility of the app for older HCW, duplication with existing apps, and responsibility for verifying and updating vaccination records, where these are administered by different providers, were raised.

*I guess if they were in electronic record, it would be useful. But I guess, if you are getting vaccinations done privately, whether that would still add to that record?* HCW28, 1 dose.

*It sounds like a good plan, but you do kind of get a bit swamped in apps. So, I do not know. I have got the NHS app, I guess if I wade far enough back would it be on there? All of my Covid vaccines show up on there. So, if it’s duplicating it’s not helpful but if it’s not then it’s helpful.* HCW19, Unsure.

*The disadvantages are I’m not sure I would know how to manage it. I’m sure it could be taught. How secure would it be? And does it matter if it’s not secure? Yes, it does, because if you have got something that you choose not to disclose, can someone access that information without your permission? So, I think that would be an issue for me.* HCW10, 1 dose.

#### 3.2.2 Lack of perceived importance of measles vaccination

The low perceived risk posed by measles and lack of emphasis on measles immunity in pre-employment screenings suggested a lack of importance of measles vaccination to some, particularly when contrasted with the emphasis that was seen as being placed on other infections such as hepatitis B or TB.

*As far as I remember, there’s no clear kind of a list that they give and see, “Okay, have you had these vaccines? And then if not, then you need to do this.” So it was, kind of like all over the place. So, when we came in, the only thing, like I said, they were looking at was the TB. So, we provided them with the chest x-ray and the TB certificate from back home, and that was it.* HCW17, Unsure.

*When I saw your email, I never knew that screening for measles was part of the screening process. I remember clearly hepatitis B and I think that may have been part of the screening I did as a student […] but I do not ever recall there being screening for measles. […] I cannot remember that I was ever offered screening or testing for measles.* HCW12, Unsure.

*I remember completing an occupational form, and I’m sure there was a question about vaccination. But I did say, “I’m not sure.” But I’ve never been followed up or something like that. […] So, I think I was sent a form to complete, sent the form back, and that’s that. I never had a one-to-one or anything like that.* HCW16, Unsure.

## 4 Discussion

Our study provides insights into the barriers and facilitators to measles vaccination amongst a sample of HCW, and reasons why many do not know their vaccination status. HCW in this sample expressed generally positive attitudes toward vaccination; however, some had reservations. MMR vaccination decisions were informed by a process of weighing up the “*pros and cons*” (HCW21, Unsure). Many of the barriers and facilitators identified in this study reflect existing findings. Although not specific to measles vaccination, a systematic review (34) found that vaccine hesitancy in HCW may be compounded by similar individual (e.g., concerns about vaccine safety or efficacy, a lack of knowledge of the vaccine and vaccination, distrust of pharmaceutical companies and assumed immunity) and structural factors (e.g., time constraints and costs). Meanwhile, Thompson and colleagues (35) reported that MMR vaccine uptake in England was impacted by poor awareness of the risk of measles, a lack of trusted information, mis/disinformation, personal experience, concerns about vaccine safety and effectiveness, population mobility, service availability, inequalities in subpopulations, affordability, and accessibility. Existing models suggest that vaccine hesitancy is driven by convenience, complacency, confidence (the ‘3c’ model), and, latterly, calculation (active seeking of information on the risks and benefits) and collective responsibility (5c model) (36, 37). The barriers and facilitators identified in previous and the current study map onto these dimensions of vaccine hesitancy.

Accessibility, as an indicator of convenience, was a recurring barrier to vaccination. Considering the workload and time constraints under which HCW operate, there is a need to simplify access to vaccination by offering flexible times and locations, and access to free vaccines. There is some evidence that increased accessibility has a positive impact on influenza vaccination (38). Low perceived risk of measles and fear of potential vaccine side effects can impact complacency and confidence. Both may be compounded by gaps in HCW knowledge about potential health risks of measles and the benefits of measles vaccination (39). Participants in the current study reported limited knowledge of measles and the vaccine, and many would want to know more before agreeing to vaccination. Few recalled measles campaigns or receiving measles-related information at work, which may impact their perception of the risk of the disease and the need for vaccination. There is evidence of lower COVID-vaccine hesitancy among Italian HCW when assessed during than before a COVID vaccination campaign (8.9% vs. 18.2%) (40). While increasing knowledge alone may not be sufficient in the absence of structural changes which also improve access, future efforts involving HCW-focused educational campaigns are warranted. These may require a multi-modal approach. For instance, information could be disseminated online (e.g., on staff websites and emails), physically (e.g., posters and leaflets), and verbally (e.g., in specific staff training and/or measles champions attending staff meetings) to maximize the visibility of the campaign amongst individuals with severe time constraints and different information-access preferences.

Facilitators to measles vaccination identified in this study included protection, being prompted, and pragmatic considerations. Self-protection and collective responsibility to protect others have been identified as motivators for vaccination among HCW elsewhere (41, 42). A sense of responsibility to protect patients may be particularly pertinent among HCW whose role it is to care for patients. The 5c model of vaccine hesitancy posits collective responsibility as one of five psychological antecedents of vaccination (37), suggesting that information about the role of vaccination in protecting others may be helpful in campaigns. There is some evidence that proactively identifying and inviting potentially susceptible adults, including migrants, for vaccination increases vaccine uptake, combined with reminders for boosters and checks amongst a busy workforce (43, 44).

Most participants in this study did not know their vaccination status due to a lack of access to or availability of records. This highlights a need for a central, easy-to-access portal where vaccinations administered by GPs, hospitals, pharmacies and through private healthcare are recorded. Support for an App or portal where vaccination information is stored was high, particularly where this included a function to send reminders for boosters.

Many participants did not recall that measles formed part of the OH screening. Others described no follow-up to update or replace misplaced records. There may be scope to place greater emphasis on establishing immunity, updating records and informing HCW of their vaccination and/or immunity status during the screening to underline the importance of measles protection. Studies assessing measles vaccine uptake following immunity testing and referral of seronegative HCW and medical students for vaccination showed that between 47.7% and 95.9% agreed to vaccination (45–47). This suggests that information about one’s susceptibility may be a useful prompt for vaccination. Previous work has demonstrated that only a third (31%, *n* = 25/80) of NHS OH departments were found to screen for measles immunity at the pre-employment stage (26). Sixteen of these took no further action when there was no evidence of immunity, while nine recommended immunization in-house (*n* = 5) or through the general practitioner (*n* = 4). Moreover, 20 relied on history alone, which may not be sufficient considering the proportion of HCW who are unsure of their status.

Finally, pragmatic considerations such as believing there is a requirement to be vaccinated for work may facilitate vaccination. However, vaccine mandates, although effective, are controversial (48, 49). HCW in our study highlighted the importance of enabling people to make an informed decision about vaccination, by disclosing vaccine requirements at the recruitment stage, providing sufficient information about measles and the vaccine prior to any vaccination appointments and engaging HCW in conversations about vaccinations to allow them to ask questions and voice their concerns.

Psychological models such as the 3c and 5c models (36, 37) and the COM-B model (50) may be usefully applied to identify possible interventions. In the COM-B model, behavior is proposed to arise from the interaction of capability, opportunity, and motivation (50). Those who have concerns about vaccine safety, assumed immunity and a lack of awareness of the risks of measles may have little motivation to be vaccinated, whilst structural factors such as time constraints, access and costs may limit the opportunities for HCW to become vaccinated. Increasing knowledge of measles and the measles vaccine, and one’s own susceptibility through immunity testing, may help to decrease complacency and increase confidence (or motivation), while improved accessiblity and prompts may increase convenience and opportunities. Moreover, interventions could include information about the role of HCW in keeping their patients and loved ones safe.

**Figure 2:**
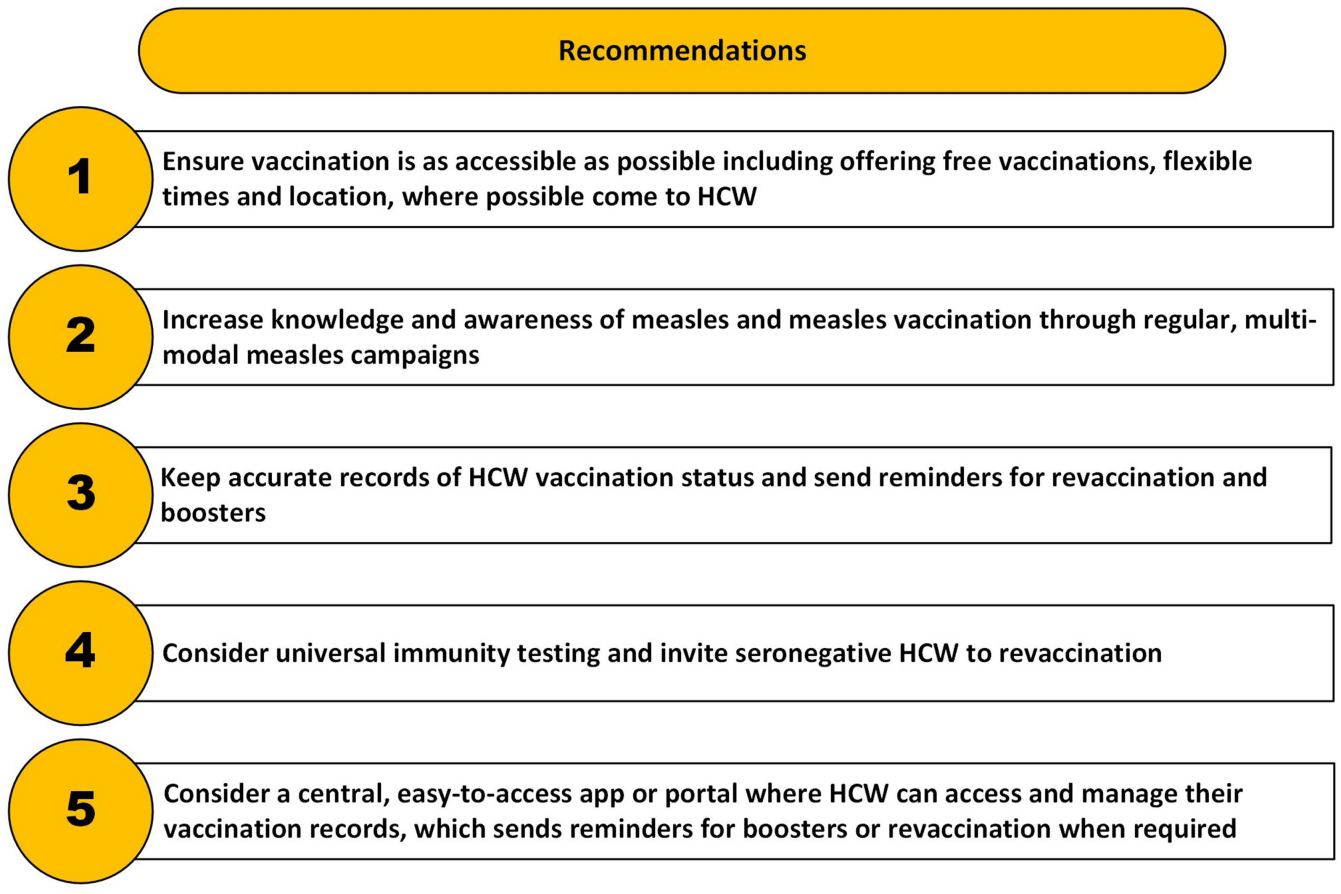
Summary of measures to support measles vaccination in HCW and reduce the number who are unsure of their vaccination status.

Overall, the findings suggest that the measures summarized in Figure 2 may help those who hold positive or equivocal attitudes toward vaccination complete or start their measles vaccination schedule and reduce the number of HCW who are unaware of their status.

### 4.1 Limitations and future research

The suggestions outlined in Figure 2 are preliminary due to several limitations associated with this study. First, this study consisted of a self-selected sample of HCW recruited from a single hospital, where institutional culture and vaccination policies may not be representative of broader systems. All participants were female and most were nurses. Several HCW from the same teams may have taken part due to the use of convenience sampling. Three men contacted the research team but were ineligible to take part. The findings may therefore not reflect those of male and other healthcare professionals. Only two participants were unvaccinated, one of whom conceded that her mother may have taken her to be vaccinated without telling her. Both had positive attitudes toward vaccination. This means that the views of those who are strongly against vaccination are not represented here. Second, self-reported vaccination status could not be verified due to the difficulty in obtaining vaccination records discussed in this article and to ensure HCW anonymity. Third, despite our efforts to emphasize the confidential nature of the research, some participants may have been reticent to be fully open about their perceptions toward vaccination or their employer’s support. We may not have uncovered the full range of challenges that staff face when considering or accessing vaccination. Quantitative methods may provide greater confidentiality, and future research could use these to confirm our findings in a larger, representative sample. Qualitative work in other trusts could confirm if the experiences expressed in study apply across healthcare settings.

## 5 Conclusion

This study provides insights into the barriers and facilitators to measles vaccination among HCW in a single UK hospital in the context of recent measles outbreaks across the UK. Future efforts should focus on vaccination prompts and programs to improve knowledge about the vaccination and the disease itself amongst HCW. Our findings support the need for ensuring ease of access identified in existing evidence. Better systems are also needed to track and maintain vaccination records to allow identification of susceptible individuals during an outbreak. Developing and implementing ways for HCW to have autonomy over their own vaccination records, such as using digital tools that could include an easy-to-access App or portal that sends reminders for boosters or revaccination when required, may reduce the number of HCW who are unsure of their vaccination status.

## Data Availability

Where consent was given, the pseudonymised transcripts presented in this study can be found in online repositories. The names of the repository/repositories and accession number(s) can be found at: King’s College London Open Research Data System (KORDS), https://kcl.figshare.com/.

## Author disclaimer

The views expressed are those of the author(s) and not necessarily those of the NIHR, UKHSA or the Department of Health and Social Care. For the purpose of open access, the author has applied a Creative Commons Attribution (CC BY) licence to any Author Accepted Manuscript version arising.
