# Supplementary material for: A qualitative interview study exploring barriers and facilitators to uptake of measles vaccination among healthcare workers at a London hospital
Source: Front Public Health. 2025 Sep 26;13:1621699. doi: 10.3389/fpubh.2025.1621699 (PMC12511023; doi:10.3389/fpubh.2025.1621699)
Supplement: Supplementary file 1 [file Data_Sheet_1.pdf]

Screening questionnaire

Eligibility questions:

Q1 How old are you today?
Age ranges – **Exclude if aged <18**

Q2 What is your current role at [the Trust]?
Type in

Q3 In your role, do you have direct contact with patients?
1. Yes
2. No – **Exclude**

Q4 Have you been diagnosed with measles in the past?
1. Yes – **Exclude**
2. No
3. Don't know

Q5 Which of the following statements best describes your measles vaccination status?
1. I am not vaccinated against measles
2. I am not sure if I have been vaccinated against measles
3. I have received one dose of the MMR vaccine
4. I have received both doses of the MMR vaccine

IF Q5 = 3 or 4

Q6 Did you receive one or both doses of the MMR vaccine after joining KCH?
1. Yes
2. No – **Exclude**
