# Supplementary material for: A qualitative interview study exploring barriers and facilitators to uptake of measles vaccination among healthcare workers at a London hospital
Source: Front Public Health. 2025 Sep 26;13:1621699. doi: 10.3389/fpubh.2025.1621699 (PMC12511023; doi:10.3389/fpubh.2025.1621699)
Supplement: Supplementary file 2 [file Data_Sheet_2.docx]

Supplementary Material

# Draft interview schedule:

Q1. To start off, can you tell me a little bit about what you do at [*Trust*]?

Q2. Moving on to measles now, could you tell me a little bit about your personal and professional experience with measles.

*Prompts:*

1. Have you come across measles in your work (patient) or personal life (family member)?
2. Do you know if you have had measles in the past?

*Probes:*

1. How likely do you think you are to be exposed to measles at work in the next few years? Why?
2. If you caught measles, how worried would you be? Why?

VACCINE STATUS AND SCREENING

Q3a. In our initial call, you told me that you are not vaccinated/ don’t know if you are vaccinated/ had only received one dose of the vaccine/ were not vaccinated before joining [*Trust*].

How confident are you that you are/were not vaccinated? How come?

*Probes*:

1. Where did you check your vaccination status?
2. Have you been asked about your vaccination status in previous healthcare roles?
3. Have you had a blood/saliva test to see if you are immune?
4. Some healthcare organizations require evidence of vaccination or other immunity. How do you provide evidence of your immunity?

Q3b. Can I just check why you are not vaccinated against measles? / why you decided to be vaccinated after joining [*Trust*]?

*Probes*:

1. What are your main concerns/motivations?

Q4. Have you tried to find out your vaccination status in the past?

*Probes*:

1. What did you do? Why not?
2. Do you know if you are immune?
3. Have you been asked about your vaccination status in previous healthcare roles?
4. Would you like to know your vaccination status?
5. How would you like to review your vaccination status?

Q5. I understand that healthcare staff go through a screening process to find out their vaccination status for diseases such as measles when they first start working at [*Trust*]. Could you talk me through your experience of the screening process?

*Prompts*:

1. What happened – talk me through it.
   1. When were you first contacted about this?
   2. Who contacted you?
   3. What was done? Questionnaire vs tests?
   4. What information was provided at each point?
   5. Was there any follow-up?
   6. How were appointments for catch-up vaccines set up?
2. How did you find it? How easy did you find the whole process?
3. If [*Trust*] was redesigning the screening process, what could they do to make the process better?

REASONS FOR (NON-)VACCINATION

Q6. I would now like to go back to the measles vaccine. I’m curious to hear your views on the measles vaccine. How do you feel about the MMR vaccine?

*Probes*:

1. To what extent do you think it is effective?
2. How safe do you think it is?
3. Does this reflect your view of vaccines in general?

Q7. How much do you feel you know about the MMR vaccine?

*Probes*:

1. What would you like to know more about?
2. Who or where do you go for information about vaccines that you trust?
3. How do you decide if you can trust a source or not?
4. How would you like to receive information about the MMR vaccine?

Q8. If there was an outbreak of measles at [*Trust*] and you were asked to have the MMR vaccine, would you have the vaccine? Why/why not?

*Probes*:

1. What are your main concerns / reasons?
2. Is there anything that you have doubts about / makes you think maybe?
3. Would you consider having the vaccine if your family or your colleagues asked you to have it?

Q9. If you decided you wanted to get vaccinated, what would help you have the MMR vaccine?

*Prompts*:

1. Where and when would you like to receive the MMR vaccine?
2. Who would you like to give you the vaccine?
3. How accessible is MMR vaccination at [*Trust*]? Is it free?

*Probes*:

1. Would you consider having the vaccine if it was easier to get it?

INTERVENTIONS

Q10. Are you aware of any recent measles campaigns at [*Trust*]? Could you describe them to me.

*Probes*:

1. What did you think about the campaigns?
2. How did they make you feel about the MMR vaccine?
3. How useful did you find the information included in the campaigns?
4. How trustworthy did you find the information in the campaigns?
5. What would be helpful to do in future campaigns to help people decide if they want to have the vaccine and what should be avoided?

Q11. This isn’t something that is being considered in the UK, but in some countries, the MMR vaccine is mandatory for healthcare workers. How do you feel about that?

*Probes*:

1. Is there any time or location where you would think this would be acceptable?

Q12. There has been some conversation about an app where healthcare workers’ vaccination status for various diseases can be recorded. How would you feel about this?

CLOSING

Before we close, is there anything else that you would like to tell me about measles, the MMR vaccine, or the process at [*Trust*]?
